# A cluster randomized implementation trial to measure the effectiveness of an intervention package aiming to increase the utilization of skilled birth attendants by women for childbirth: study protocol

**DOI:** 10.1186/1471-2393-14-109

**Published:** 2014-03-19

**Authors:** Gajananda P Bhandari, Narayan Subedi, Janak Thapa, Bishnu Choulagai, Mahesh K Maskey, Sharad R Onta

**Affiliations:** 1 Nepal Public Health Foundation, Post Box 4004, Kathmandu, Nepal; 2 Institute of Medicine, Tribhuwan University, Kathmandu, Nepal

## Abstract

**Background:**

Nepal is on track to achieve MDG 5 but there is a huge sub-national disparity with existing high maternal mortality in western and hilly regions. The national priority is to reduce this disparity to achieve the goal at sub-national level. Evidences from developing countries show that increasing utilization of skilled attendant at birth is an important indicator for reducing maternal death. Further, there is a very low utilization during childbirth in western and hilly regions of Nepal which clearly depicts the barriers in utilization of skilled birth attendants. So, there is a need to overcome the identified barriers to increase the utilization thereby decreasing the maternal mortality. The hypothesis of this study is that through a package of interventions the utilization of skilled birth attendants will be increased and hence improve maternal health in Nepal.

**Method/Design:**

This study involves a cluster randomized controlled trial involving approximately 5000 pregnant women in 36 clusters. The 18 intervention clusters will receive the following interventions: i) mobilization of family support for pregnant women to reach the health facility, ii) availability of emergency funds for institutional childbirth, iii) availability of transport options to reach a health facility for childbirth, iv) training to health workers on communication skills, v) security provisions for SBAs to reach services 24/24 through community mobilization; 18 control clusters will not receive the intervention package. The final evaluation of the intervention is planned to be completed by October 2014. Primary study output of this study is utilization of SBA services. Secondary study outputs measure the uptake of antenatal care, post natal checkup for mother and baby, availability of transportation for childbirth, operation of emergency fund, improved reception of women at health services, and improved physical security of SBAs.

**Discussion:**

The intervention package is designed to increase the utilization of skilled birth attendants by overcoming the barriers related to awareness, finance, transport, security etc. If proven effective, the Ministry of Health has committed to scale up the intervention package throughout the country.

**Trial registration number:**

ISRCTN78892490.

## Background

Millennium development goals for the improvement of maternal health has shown suboptimal achievements in less than one fourth of countries where it is on track to reduce maternal mortality ratios (MMR) by 3/4^th^ (below 139/100000 live births) [1]. Only 16 of 68 countries are on track to achieve 2/3^rd^ reduction in under-five mortality by 2015 [2,3]. Increasing skilled attendant at birth is an important indicator for ensuring better maternal, child and neonatal health [4].

Nepal is making considerable progress in achieving Millennium Development Goals (MDG) 4 and 5 [1,3,5]. However, progress is uneven at sub-national level, between developmental and ecological regions, and in populations of different socio-economic status [6,7]. The national average of Infant Mortality Rate (IMR) per thousand live births is 48, but mid-western and far-western districts have 58 and 65 respectively. Likewise,mountain districts have the higher IMR (73) as compared to hill and terai districts (50 and 53) as per Nepal Demographic and Health Survey (NDHS) 2011 [8].Similar patterns are observed for MMR [9].

Despite a decrease in maternal mortality (770 in 1990 to 170 in 2010), [10] the underlying causes of maternal deaths remain of serious concern. An analysis of maternal deaths [1] reveals that nearly 83% of all maternal death is attributable to direct causes: the three main causes being post-partum hemorrhage (32%), hypertensive disorders during pregnancy (25%) and abortion (13%) [11]. Two third of infant deaths occur during the neonatal period [12]. Without improved availability and utilization of skilled birth attendant’s (SBAs) to address the direct causes of maternal deaths, further reduction in maternal and neonatal mortality is extremely unlikely. Amidst low SBA service coverage at the time of birth (28.8%) against the target to be achieved by the year 2010 (50%) and 2015 (60%) [13] women are still facing barriers in utilization of SBA services in Nepal. Research is needed to explore [1] the approach that will be cost effective and scalable to overcome the barriers to utilization of skill birth attendant services in Nepal.

### Efforts of national health system for maternal and child health improvement

In response to the need for accelerated actions to reach the desired reductions in maternal and newborn mortality, the government of Nepal (GoN) has devised several policy strategies and programs [14]. The National Health Policy (NHP) formulated in 1991 with the objective of enhancing the health status of rural population extends the primary health care system with the intention of increased access to modern medical facilities and trained health care providers.The subsequent health plans were based on the NHP and focused on strengthening neonatal, child and maternal health in Nepal. These include the Eighth Health Plan (1992–1997), the Ninth Health Plan (1997–2002), Tenth Five Year Plan (2002–2007), Three Year Interim Plans (2008–11, 2011–13) and the Second Long Term Health Plan (SLTHP) (1997–2017). The Three year interim plans aimed to provide free essential health care services for all citizens and the safe delivery incentive programme to accelerate the country’s progress towards achieving Millennium Development Goals 4 and 5 [15,16]. In these plans, achieving health results in women, children and vulnerable, marginalized, disadvantaged populations, as well as those living in hard to reach areas have been prioritized. The primary healthcare approach is central to the delivery of services [8].

The Safe Delivery Incentive Programme (SDIP), started in 2005, is central to government’s effort to improve maternal health. It aims to increase coverage of skilled birth attendance and also to contribute towards poverty reduction by preventing death and disability [17,18]. The SDIP was developed to help households overcome financial barriers to accessing maternity care. According to the revised policy guidelines, the SDIP provides: Women giving birth at eligible government health institutions (hospital, primary health care centre, or health post), medical colleges, mission hospitals and NGO-run hospitals receive a fixed sum of 1,500 NRs ( 20 $) in mountain areas, 1,000 NRs (12 $) in hill areas, and 500 NRs (6$) in the Tarai [19]. But, failure to pay the financial incentives to the women on time in many districts has resulted in low acceptance of the offer of the cash incentive. Consequently, low SBA coverage continues despite the financial incentives [20].

The GoN has endorsed a SBA policy in 2006 within the framework of the Safe Motherhood Policy 1998, and aims to increase service coverage to 60% by the year 2015 [21]. While the international target of SBA coverage is 90%, the target set by Nepal is in line with the WHO target for the countries with high levels of maternal mortality [21,22].

The progress in meeting this target, however, is slow, as SBA coverage has reached only 28.8% by 2010 [23]. This coverage accounts for service provided by ‘any’ kind of trained health professionals, many of which do not meet the strict criteria set by the SBA policy of Nepal [21] This SBA coverage would mainly refer to this mix of health professionals who may or may not be trained as SBAs. This is to happen unless SBA training to the health professionals is expanded quickly and in massive scale.

The government has deployed three major strategies; promoting birth preparedness and complication readiness, institutional childbirth, and expansion of 24-hour emergency obstetric care through the Safe Motherhood and Neonatal Health Long Term Health Plan (SMNHLTP 2006–2017). The SMNHLTP also includes an increased focus on other areas like the importance of addressing neonatal health as an integral part of safe motherhood programming, SBA policy, health sector reform initiatives, legalization of abortion and integration of abortion into the safe motherhood umbrella, prevention of mother to child transmission and emphasis on needs of poor and disadvantaged women [17].

In view of the low coverage of skilled birth attendance in Nepal, the Nepal Public Health Foundation baseline survey explores the barriers to seeking care with skilled attendants for childbirth care [24-27]. The findings showed that the cost of institutional birth, including the cost of transport, unavailability of transportation systems, insecurity of SBAs at health facilities discourage utilization of SBA services by women [28].

It is to be noted that even in terms of child birth assistance from a health personnel, Nepal ranks lower in South Asia. While Nepal and Pakistan have similar coverage (19%) ranking the second lowest, Bangladesh has lowest 13% and Sri Lanka has the highest 96% of births delivery attended by health personnel [8].

Our project baseline during April to December 2012 in Bajhang, Dailekh and Kanchanpur districts of Mid and Far Western Development region in collaboration with GoN and financial and technical support form WHO Geneva reported first ANC coverage of 88% while 4^th^ and more ANC visit of 65% in average. Only the half of the respondents (52%) knew about danger signs during pregnancy and delivery and 19% of them had faced complications during last delivery.

Our survey reported, 45% of births were assisted by SBA/THW which ranges from a high of (64%) in Kanchanpur and a low of (33%) in Bajhang. Which is low as compared to MDG target to scale up SBA coverage to 60% by 2015 [13]. This suggests additional efforts are required in scaling up skilled birth attendants in Nepal. This requires innovative intervention that uniquely address identified barriers in receiving care. Distance to reach health facility, lack of transportation means to reach the health facility, lack of information about the availability of services in health facilities were the identified barriers.

Amidst of possibilities and challenges in SBA service scale up, Nepal Public Health Foundation (NPHF) [24] has taken initiative and has been conducting a study on “Overcoming Barriers to Scaling Skilled Birth Attendants Utilization in Improving Maternal, Newborn and Child health in Nepal” as a three-year implementation research project with support from WHO-HQ, Geneva. The overall aim of the implementation research is to increase SBA service utilization by identifying the barriers related to service users and health systems regarding the utilization of skilled birth attendants and implementing a package of interventions to address these barriers.

### Hypothesis

It is hypothesized that through a package of interventions which includes activities to increase family support for pregnant women to reach health facility, make available funds to remove financial barriers faced by families for using institutional childbirth care, make available transport options to reach a health facility for childbirth, improving providers’ communication skills, and reduce security problems of SBAs so that care can be available 24/7 will lead to increased utilization of the services of a skilled birth attendant and subsequently improvements in maternal, newborn and child health in districts of Far and Mid West Nepal.

### Study objectives

#### ***Primary research objectives***

The primary objective of this study is to increase the utilization of SBA services for childbirth.

### Secondary research objectives

1. To increase the utilization of four antenatal care visits by pregnant women and postnatal care services by mothers and babies.

2. To evaluate the effectiveness of the proposed implementation package in increasing SBA service utilization for childbirth care.

3. To assess the feasibility and scalability of the implementation package in other districts of Nepal.

## Methods/Design

### Study population

The intervention is being implemented in three districts of mid and far-western Nepal. Among them, one district is located in the mountains (Bajhang), one in a hilly zone (Dailekh) and the other in the plain terai (Kanchanpur) zone. Bajhang, Dailekh and Kanchanpur respectively have 48, 54 and 20 Village Development Committees (VDCs). The VDCs are the basic politico-administrative unit of Nepal which is further divided into nine smaller units called Wards having an average of 150 households (less in mountains and more in Terai) [29]. Each VDC consists of at least one health institution [17,30]. There are about 4000 VDCs in Nepal, out of which about 215 have Primary Health Care Centres, 699 have health posts (HP) and 3100 have sub health posts (SHP). According to the Nepal SBA policy, the health posts serve as the first contact birthing center for institutional birth and all the SHPs are being upgraded to HPs.

### Study design

The implementation research underway is to test the impact of interventions designed to address the main barriers to utilization of SBA services at birth identified in the baseline study. The study design is a cluster randomized controlled trial. The three districts (Bajhang, Dailekh and Kanchanpur) were the three strata and the VDCs from each stratum serve as clusters which were allocated for intervention and control by *randomization*. A baseline survey was conducted in 2011 in all 50 VDCs of the three districts. All women with under-5 children from each VDCs were included.

The three districts of Far and Mid-west Nepal represent three ecological zones –- Kanchanpur is Terai or a lowland belt stretching east to west in the south; Dailekh is Hills; and Bhajang is a mountainous areas. Selection of three districts was based on ecology with low human development index. The population inhabiting these districts is varied, representing different ethnic groups [31]. Terai district is mainly inhabited by aboriginal population of Nepal the Tharus; the mid hills by Kshetri and Bahun (Aryan upper cast but lower economic status). The mountain district has predominantly Mongoloid populations. These districts have lower indicators with respect to education, health and economic development.

**Figure 1:**
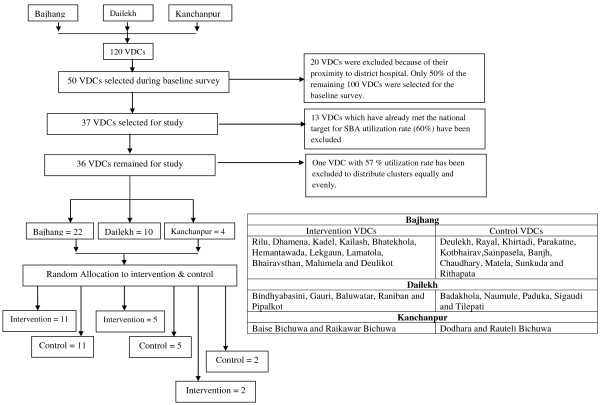
Selection of study clusters.

The implementation of intervention covers 36 of the 50 VDCs of the three districts (22 VDCs of Bajhang, 10 VDCs of Dailekh and 4 VDCs of Kanchanpur (see table in Figure 1). VDCs having a rate of utilization of SBA at birth higher than the national target of 60% were excluded from the intervention. In order to have an even number of clusters, one more VDC with coverage of 57% was excluded.

The estimated number of pregnant women covered by the intervention is around 4800 which include approximately 110 pregnant women from each VDC of Bajhang and Dailekh as a mountain and hill district respectively inhabiting less number of population, and approximately 310 pregnant women from Kanchanpur as a terai district with densely-populated lowland.

### Sample size estimate

The study includes all pregnant women in the 36 VDCs. It is estimated that at least 110 births per year will occur in each VDC and that the proportion of births assisted by a SBA is 36% [7]. The sample size was estimated assuming that the intervention and control groups and the unmatched clusters were of approximately equal size. A value of k -the between-cluster coefficient of variation – to be equal was estimated in the intervention and control groups. All estimates are based on a two-tailed 5% significance level. The value of k is likely to be 0.25 on the basis of estimates of the SBA utilization rate according to the baseline survey [24]. Hence, It was estimated that on at least 110 pregnant women in each cluster with 36 clusters would be required for the period of one year to detect a 10% difference in SBA utilization rate with 80% power and 95% confidence level.

### Development of the intervention package

The findings of the baseline survey was disseminated in 2011 to all the stakeholders at the central level (focal person from Ministry of Health, Director General from Department of Health Services, Director of Family and Child Health Division) and district level (District Public Health Officer, Public Health Nurse, Focal Persons, Local Leaders, Media) by organizing a dissemination meeting at central and district level. Feedback was collected about the possible interventions to address the different barriers identified. The implementation package was also discussed with teams from the World Health Organization. The findings of the baseline study can be found in Nepal Public Health Foundation website (http://www.nphfoundation.org).

In dialogue with the representatives of Government of Nepal, Family Health Division, a model of community mobilization was developed that could be scaled up within the existing health system, which focused on enabling SBA attendance at birth, and strengthening links to be developed between communities and health facilities. An intervention package was developed that fits the national policy of engaging female community health volunteers (FCHVs), Health Facility Management Committee (HFMC), and Youth Groups to increase utilization of SBA services.

### Interventions

The intervention package has five components and a diverse set of activities which aims to overcome the barriers in scaling up SBA services in Nepal.

1. Lack of Family Support for the Pregnant Women to Reach a Facility for Childbirth.

2. Lack of Funds to for a Women and Families to Seek Childbirth Care with a SBA.

3. Unavailability of Transportation to a Facility for Childbirth.

4. Lack of Women-friendly Environment in Health Facilities.

5. Issues of Security of SBA.

A team from the Nepal Public Health foundation coordinates the activities. There are team leaders for each of the three districts. Each VDC has a research assistant responsible for proper implementation of the intervention package and on-site monitoring of all activities during the intervention period. An extensive training had been given to the district level team (includes district coordinator in district and research assistant at VDC level) to implement the intervention package.

### Component 1: Increased family support for pregnant women to reach a facility for childbirth

FCHVs were oriented by project staff to discuss with pregnant women and members of their families the importance of birth with a skilled attendant, and review with the family the birth and emergency preparedness card which the pregnant woman completes in her antenatal care visits.

There are mothers group under each FCHV in each ward. Mothers groups were given an orientation by FCHVs with the support of project staff to invite the families of identified pregnant women (identified in each ward through FCHVs and mothers group members) to regular meetings to discuss the importance of birth with a skilled attendant and family member’s support to the pregnant women to reach the health facility and to access childbirth care with a SBA. The mothers groups provide information to the family members about the emergency fund and transportation arrangement mechanisms discussed below.

### Component 2: Availability of funds to support pregnant women and families to seek childbirth care with an SBA

Currently in Nepal, mothers groups have funds which can be utilized for any health related purposes and regarded as an emergency fund. The research team organized discussions with the HFMCs of each Health Post, Sub Health Post regarding the operation of an emergency fund by the mothers groups. The discussion included the roles and scope of the HFMC to support mothers groups in operating the fund and possible modalities for its operation, particularly how to protect against losses and replenishment of the fund. The project staff supported the HFMC in their discussions with mothers groups for the expansion of the current fund, to agree upon a modality of operation and to establish regular coordination and feedback between the HFMC and mothers group. All mothers group agreed to use the existing fund as an emergency fund to support women to reach health facility which is supposed to be reimbursed by the women after getting the incentives from health institution as a part of the Safe Delivery Incentive Program in Nepal.

### Component 3: Availability of transportation to a facility for childbirth

The project staff interacted with the HFMC of all intervention VDCs to discuss the development of a system to organize transportation of mothers for reaching facilities for childbirth and in case of complications. The possibility to mobilize local actors, particularly local youth groups and mothers groups, was discussed. There was also a discussion on the modality and functioning of the system.

Project staff supported the HFMC to organize a meeting with youth groups to discuss the transport needs of pregnant women to reach health facilities for childbirth and in case of complications. The Youth groups agreed to support. Members of the HFMC periodically organize meetings with the youth groups to review the activities, discuss any problems faced and possible solutions.

### Component 4: Development of a Women-friendly environment in health facilities

The HFMC oriented the SBAs and staff on improving the friendliness of the facility for pregnant women and their families. Furthermore, training on communication skills was also given to the SBAs and staff of each facility. Meetings were organized with the health facility management committee and with providers to discuss the policy to allow a companion of the women’s choice during childbirth. SBAs were oriented on how to work with the companion during labour and birth.

### Component 5: Establishment of mechanisms to improve security of SBAs

As the security of female SBAs was identified as one of the barriers to ensuring service availability, meetings were organized with the HFMCs, mothers groups, youth groups and other relevant actors in the community to discuss the situation and agree upon the mechanisms to improve the security situation of SBAs. For arriving to the facility at night, SBAs will be accompanied by a member of the youth groups or a FCHV of the particular area of a village during childbirth.

### Measuring the impact of the intervention package

Data on the primary outcomes of the project will be collected through periodic surveillance and an end line survey (evaluation phase) in all VDCs of project. For the evaluation to begin in May 2014, Surveyors will administer questionnaires to the women in intervention and non-intervention VDCs. Qualitative research will be conducted to evaluate the process indicators. The questionnaires will be the same used during the baseline phase of project with the primary and secondary outputs indicated below. The surveyors and data analyst will be blinded for the evaluation phase.

### Primary output

Utilization of SBA services by the women for childbirth.

### Secondary output

Ante-natal care visits for 4 times by pregnant women

Post natal care visits of mothers and babies

Availability of transport to the women to visit health institutions for childbirth

Functional operation of emergency fund

Security of SBA

Family support to the women for childbirth at health institution

Women friendly health facility environment

### Project time line

Intervention preparation: October, 2012

Formation of steering committee, preparation for trainings: November to January, 2013

Meeting and training: February, 2013

System of transportation and Emergency fund establishment:February to April, 2013

Intervention:May, 2013 to April, 2014

Evaluation of project: May to October, 2014

The evaluation of the impact of intervention will be done using multiple methods. Both the qualitative and quantitative methods will be used in evaluation. End line survey to determine the effects of the interventions will be done with key process indicators. This will include questionnaire surveys with 5000 women in 36 VDCs in three districts.Health workers, female community health volunteers, youth group members, health facility management committee members will be interviewed.

The instruments used for the base line survey will be used for the evaluation of outcome. Necessary modifications will be made to the instruments.Data will be entered in Epi data and imported to SPSS for analysis. Results from the bivariate and multivariate analysis will be compared with the baseline results. Intervention and control VDCs will be compared to identify any difference in the outcome variables to find out the association of the intervention components for possible change. Test of significance will be done at 5% significance level using appropriate statistical test.

### Interim analysis

A mid-term data monitoring will be conducted during the mid-intervention phase in 2013. For this a data management committee will be formed. The data management committee will report how well the project has been implementing. They will check the key methodological component of project; whether enrolment has been adequate, whether randomization has been done. End line survey will serve as evaluation of project. The evaluation phase will have preparation of evaluation tools, recruitment of enumerators and supervisors which will help in interim data analysis. This team will also check the completeness in the implementation of intervention data, adherence to the ethical principles and will advise for the improvement in intervention phase in case of detected opportunities for improvement.

### Analysis strategies

Data will be double entered into EpiData 3.1, validated using Epi Info data compare, and will be transferred to SPSS 16 for analysis. The findings from quantitative tool will be presented in tabular and graphical forms. To control for more than one confounding variable multivariate logistic regression model will be applied. For statistical inference of the result, a confidence level of 95% is set. For the analysis process of qualitative data collected form FGD, the transcription of taped FGD session will be typed and reviewed thoroughly then translated in English language by field coordinators. The content of transcription will then be labeled as per the domain of analysis.

Missing data will be characterized in terms of the degree and patterns of missing data. For imputation of missing value of categorical variables, the frequency distribution will be used as the basis for randomly generating a value for each observation lacking a response. For example, if education was measured in three categories -- “less than high school” (25% of complete data cases), “completed high school” (40%), or “more than high school” (35%) -- then for each observation with education missing, a random number between 0 and 1 will be drawn from a uniform distribution and the missing value replaced with “less than high school” if the random number is less than or equal to 0.25, “completed high school” if the number is greater than 0.25 but less than or equal to 0.65, or “more than high school” if greater than 0.65. For imputation of numerical variable regression method is used.

### Ethical considerations

The research got the approval from Nepal Health Research Council in 2011. The informed consent forms have been developed according to the WHO and National Ethical Guidelines. Sharing meeting was organized with the district level stakeholders, management committees and mothers groups in all study clusters before randomization and shared about the research objectives, approaches and duration. We also presented findings of our baseline in graphical and pictorial presentation for ease of understanding. The committees and groups were explained by the research team that interventions will be implemented only in half of the VDCs which will be selected randomly. The committees and groups were explained about the modality of interventions.

The enumerators during the end line survey will approach participants in their homes; will take written consent before the interview. Participation of women is voluntary. Also, NPHF has made provision to make study areas safe for field coordinators and enumerators.

### Benefits to control areas

There is no direct benefit to the control VDCs. However, if the trial found to be effective, the government of Nepal has committed to scale up the intervention package starting from the control VDCs.

The intervention will not benefit the individual nevertheless it aims to strengthen already functioning health system. The intervention is not being done at the individual level rather than at the system level. These interventions are strengthening the capacity of health system by reinforcing their ongoing tasks or expanding the scope of their activities.

### Sustainability and scalability

In all intervention components, we are directly working with health care system of Nepal which increases the sustainability of our approach.We trainedfemale community health volunteers, oriented health facility committee members, local youths to actively communicate the importance of skill attendant at birth, manage transportation system in obstetric emergency, mobilize funds and improve security to SBAs and friendliness of health institutions. Every orientation in health facility was participated by community leaders, political representatives, and health personnel and women group members. An existing health system is being strengthening without adding intervention that is not feasible and acceptable. It is hoped that after the end of this intervention the communities will be self-reliant in their capacity to provide skill attendant at childbirth.

### Public engagement

The project encourages public engagement at different levels. Locally members of health facility management committee, female community health volunteers, and local youth groupswere involved starting from formative phase where they identified barriers in utilization of SBA services to design of intervention package.Locally intervention areas will have periodic meeting with community groups, women’s groups, and health facility management committee members. The filed coordinators will be present at each of these meetings every month to help them understand the importance of skill attendant at birth.

In district, regional and national level District Public Health Office (DPHO)/District Health Office (DHO), regional health directorate, district and regional governmental and nongovernmental health agencies were involved. From the very beginning,the researcher was involved in dialogue with district and national level health agencies. The project was approved by the Family Health division (MoHP) and Nepal Health Research Council. It is believed that by involving government agencies in our research workwill increase the utilization of findings of the study.

The findings of the study will be disseminated to policy makers, programme managers of governmental and nongovernmental health organization at district and national levels after completion of the end line survey. We will seek the possibility to lobby policy makers in utilizing key recommendation of our project. For this, findings will be presented to national and international conferences organized by professional bodies like Nepal Pediatric Society (NEPAS), Nepal Society of Obstetricians and Gynecologists (NESOG), Nepal Medical Association and Nepal Public Health Association (NePHA), and Tribhuvan University, Maharajgunj Medical Campus. We will also publish our findings in national and international scientific publications as well as popular papers.

## Discussion

This proposal addresses the issues of achieving MDG 2015 in Nepal. It supports the Safe Delivery Incentive Programme (SDIP) which is central to government’s effort to improve maternal health that aims to increase coverage of skilled birth attendance and also to contribute towards poverty reduction by preventing death and disability [17,18]. The protocol assesses the effectiveness of a package of community based intervention to improve maternal outcome.

The package of intervention includes five components. The first three components address the demand perspective such as increasing family support for pregnant women, making avail the fund to reach the facility and transportation. Similarly, the last two components address the provider perspective at community level such as capacity strengthening of skilled birth attendants in terms of communication training and ensuring security of the service provider.

The intervention package is designed to increase the utilization of skilled birth attendants by overcoming the barriers related to awareness, finance, transport, security etc. If proven effective, the Ministry of Health has committed to scale up the intervention package throughout the country.

## Abbreviations

ANC: Antenatal care; BEOC: Basic emergency obstetric care; DG: Director general; DHO/DPHO: District health office/district public health office; FGD: Focus group discussion; GoN: Government of Nepal; HFMC: Health facility management committee; HV: Health volunteers; IoM: Institute of medicine; IMR: Infant mortality rate; MDG: Millennium development goals; MMR: Maternal mortality rate; NDHS: Nepal demographic and health survey; NHP: National health policy; NHTC: National health training center; NPHF: Nepal public health foundation; PMTCT: Prevention of mother to child transmission; SBA: Skill birth attendant; SDIP: Safe delivery incentive package; SLTHP: Second long term health plan; SMNHLTP: Safe motherhood and neonatal health long term health plan; WHO SEARO: World health office South East Asia regional office.

## Competing interests

The authors declare there is no competing interest in this work.

## Authors’ contributions

GPB contributed in study design, implementation, prepared first draft of manuscript and final manuscript review, NS is involved in design and review of the manuscript, JT contributed during implementation and review of the manuscript, BC contributed in final review of the manuscript, MKM and SRO contributed in inception of the study including design and implementation. All the authors have contributed to preparation of draft of the manuscript. And all has read the finalized draft before publication. All authors read and approved the final manuscript.

## Pre-publication history

The pre-publication history for this paper can be accessed here:

http://www.biomedcentral.com/1471-2393/14/109/prepub
